# A Normal Reference of Bone Mineral Density (BMD) Measured by Dual Energy X-Ray Absorptiometry in Healthy Thai Children and Adolescents Aged 5–18 Years: A New Reference for Southeast Asian Populations

**DOI:** 10.1371/journal.pone.0097218

**Published:** 2014-05-21

**Authors:** Pairunyar Nakavachara, Julaporn Pooliam, Linda Weerakulwattana, Pornpimol Kiattisakthavee, Katharee Chaichanwattanakul, Racahnee Manorompatarasarn, Kulkanya Chokephaibulkit, Vip Viprakasit

**Affiliations:** 1 Division of Pediatric Endocrinology, Faculty of Medicine, Siriraj Hospital, Mahidol University, Bangkok, Thailand; 2 Clinical Epidemiological Unit, Office for Research and Development, Faculty of Medicine, Siriraj Hospital, Mahidol University, Bangkok, Thailand; 3 Division of Pediatric Infectious Diseases, Faculty of Medicine, Siriraj Hospital, Mahidol University, Bangkok, Thailand; 4 Division of Pediatric Hematology/Oncology, Department of Pediatrics, Faculty of Medicine, Siriraj Hospital, Mahidol University, Bangkok, Thailand; Baylor College of Medicine, United States of America

## Abstract

Ethnic-specific normative data of bone mineral density (BMD) is essential for the accurate interpretation of BMD measurement. There have been previous reports of normative BMD data for Caucasian and Asian children including Japanese, Chinese, Korean and Indian. However, the normative BMD data for Southeast Asian including Thai children and adolescents are not currently available. The goals of our study were 1) to establish normative data of BMD, bone mineral content (BMC), bone area (BA) and lean body mass (LBM) for healthy Thai children and adolescents; aged 5–18 years measured by dual energy X-ray absorptiometry (DXA, Lunar Prodigy) and 2) to evaluate the relationships between BMD vs. age, sex, puberty, weight, height, calcium intake and the age of menarche in our population. Gender and age-specific BMD (L2-4; LS and total body; TB), BMADLS (apparent BMD of the lumbar spine), BMC (L2-4 and total body), BA (L2-4 and total body) and LBM were evaluated in 367 children (174 boys and 193 girls). All parameters increased progressively with age. A rapid increase in BMD, BMC and BMADLS was observed at earlier ages in girls. Gender and Tanner stage-specific BMD normative data were also generated. The dynamic changes of BMD values from childhood to early and late puberty of Thai children appeared to be consistent with those of Caucasian and Asian populations. Using a multiple-regression, weight and Tanner stage significantly affected BMDLS, BMDTB and BMADLS in both genders. Only in girls, height was found to have significant influence on BMDTB and BMADLS. The positive correlation between BMD and several demographic parameters, except the calcium intake, was observed. In summary, we established a normal BMD reference for Thai children and adolescents and this will be of useful for clinicians and researchers to appropriately assess BMD in Thais and other Southeast Asian children.

## Introduction

Peak bone mass attained during childhood and adolescence is a major determinant of bone health in adults [1]. Children with certain conditions including osteogenesis imperfecta, immobilization, thalassemia, HIV and those who receive prolonged glucocorticoid treatment are at risk of developing osteoporosis [2], [3], [4], [5], [6]. Early detection and accurate assessment of low bone mass in these children can lead to early and appropriate intervention.

Several techniques for bone mineral density (BMD) measurement are currently available. One of these modalities, a dual Energy X-ray Absorptiometry (DXA) is widely used as the method of choice because of the relatively low radiation exposure, short scanning time, and its precision and accuracy [7], [8]. Variations of BMD measurement techniques undoubtedly affect values of BMD parameters. Moreover, ethnic difference also affects childhood BMD values. Failure to use appropriate BMD reference values to compare and calculate appropriate Z-score (standard deviation score) may result in an under- or over- diagnosis of osteopenia and/or osteoporosis [9]. Therefore, ethnic-specific normative BMD data using a similar BMD measurement technique should be used for accurate BMD interpretation. There have been previous reports of normative BMD data for children of Caucasian [10], [11], [12], [13] and Asian populations including Japanese, Chinese, Korean and Indian [14], [15], [16], [17]. However, the normative BMD data for Southeast Asian including Thai children and adolescents are not currently available.

Therefore, our objectives of the present study were 1) to develop normative BMD, apparent BMD of the lumbar spine (BMADLS), bone mineral content (BMC), bone area (BA) and lean body mass (LBM) reference data for Thai children and adolescents aged 5–18 years and compare these parameters with reported BMD references and 2) to evaluate the relationships between BMD vs. age, sex, puberty, weight, height, calcium intake and the age of menarche in our population.

## Subjects and Methods

### Subjects

381 healthy children and adolescents aged 5.5 to 18.8 years were enrolled in the study. Subjects were randomly selected from 11 primary and secondary schools in the urban Bangkok. The study was approved by the ethics committee of the Faculty of Medicine, Siriraj Hospital, Mahidol University. Written informed consent was obtained from patients and/or their parents before enrollment. The study was conducted in accordance to the Declaration of Helsinki and with Good Clinical Practice Guideline.

### Methods

Each subject's weight and height were measured using standard weight scale (Tanita, Illinois, USA) and Harpenden Stadiometer (Holtain Limited, Crymych, UK), respectively. Subjects whose weight or height was above (>97^th^ percentile) or below (<3^rd^ percentile) the standard growth curve for Thai children were excluded [18]. Height and weight Z-scores were calculated using Thai national standard [18]. Body mass index (BMI) was also calculated. Detailed medical history was obtained from subjects or from their parents. Children who had chronic diseases and those who received medication that could affect bone mineral density such as glucocorticoids, L-thyroxine, calcium supplementation, etc. were excluded. By these exclusion criteria, 367 children (174 boys and 193 girls) were recruited. Calcium intake was determined via a detailed food questionnaire in Thai language [19]. In children aged <10 years, the questionnaire was reviewed together with parents or guardians. The pubertal status of each subject was assessed by the same physician (P.N.) according to the Tanner classification.

BMD, BMC and BA of the lumbar spine (L2-4; LS) and of the total body (TB) were measured by Dual Energy X-ray Absorptiometry (DXA, Pediatric Software, Lunar Prodigy, Lunar Corp., Madison, WI, USA). Data were analyzed by Prodigy enCORE software (version 7.53, standard scan mode). When measuring BMDLS, each subject was positioned supine, and the physiological lumbar lordosis was flattened by elevation of the knees. All measurements were performed and analyzed by the same person (L.W). Quality assurance was performed daily. The coefficient of variations (CVs) were evaluated by duplicated measurements in 30 volunteer subjects and revealed the CVs of 0.8% for L2-4 BMD (BMDLS) and 0.7% for total body BMD (BMDTB).

Since BMD, measured by DXA, is a function of bone size and the bone mineral content (BMC) within a fixed volumetric density, BMD could be underestimated in short children. To account for differences in bone sizes or height, apparent BMAD of the lumbar spine (BMADLS) was calculated by using the model BMAD = BMD × [4/(π × width)]. Width was the mean width of the 2^nd^ to 4^th^ lumbar vertebral body. The shape of lumbar vertebral body was assumed to be cylindrical [20].

### Statistical analysis

The sample size of each age group (n) for boys and girls was calculated by using the formula: n =  (Z^2^δ^2^)/d^2^ (Z = 1.96, δ =  estimated standard deviation, d  =  distance from mean to limit). A two-sided 95% confidence interval for mean of BMD values was used. The standard deviation and the distance from mean to limit values were assumed based on the previous published normal BMD reference [10]. The calculated sample size should be at least 10 for each age group.

The mean and standard deviation (SD) of BMD of the LS and TB (BMDLS and BMDTB), BMADLS, BMC of the LS and TB (BMCLS and BMCTB), BA of the LS and TB (BALS and BATB) and LBM values were calculated for each age group in boys and girls. The mean and SD of BMDLS and BMDTB values were also calculated for Tanner stage in boys and girls. The mean and SD of daily calcium intake were also calculated. The unpaired t-test was used to determine differences in these parameters between boys and girls of the same age group or Tanner stage.

The best models for the relationships between age vs. BMDLS, BMDTB, BMADLS, BMCLS, BMCTB, BALS, BATB and LBM were chosen by regression analysis. The coefficients of determination (R^2^) were calculated. After assessment of different models, the cubic model (with the factors age, age^2^ and age^3^) and the quadratic model (with the factors age, age^2^) were found to be the best fit for girls and for boys respectively. The relationships between weight, height and Tanner staging vs. BMD and BMADLS adjusted for age were assessed by multiple regression analysis.

One-way analysis of variance (ANOVA) with post-hoc test using Bonferroni correction was used to compare the difference in BMD and BMADLS among Tanner stages for each gender.

Pearson's correlations were used to calculate the correlations between daily calcium intake and BMDLS, BMDTB and BMADLS. A *P* value of <0.05 was considered to be statistically significant.

## Results

### Anthropometric data for different age groups

The anthropometric data including height, height z-score, weight, weight z-score and body mass index (BMI) for boys and girls are shown in Table 1. The mean height Z-scores of boys and girls were 0.2±0.9 and 0.2±1.0, respectively. The mean weight Z-scores of boys and girls were both 0.2±0.8. The mean BMI values were 17.8±2.6 and 17.8±2.8 kg/m^2^ for boys and girls, respectively.

**Table 1:** Anthropometric parameters (Height, Height Z-score, Weight, Weight Z-score and BMI) of Thai children and adolescents (174 boys and 193 girls) for each age group presented as mean±standard deviation (SD).

Age (yrs)	n	Height (cm)	Height Z-score	Weight (kg)	Weight Z-score	BMI (kg/m^2^)
**Boys**						
5–6	12	110.9±4.3	−0.2±1.0	19.5±3.3	0.0±0.2	15.7±1.6
6–7	10	116.1±3.7	−0.3±0.7	20.6±2.0	0.0±0.1	15.3±1.1
7–8	12	122.5±5.0	−0.2±0.9	23.3±3.5	0.0±0.6	15.5±1.5
8–9	12	128.6±6.2	0.2±1.3	27.3±6.2	0.3±0.9	16.4±3.1
9–10	14	134.9±5.3	0.4±1.0	30.3±5.0	0.3±0.7	16.6±2.6
10–11	6	138.7±4.4	0.3±0.6	32.6±7.2	0.2±0.8	16.9±3.0
11–12	10	145.7±5.9	0.4±0.9	39.6±9.5	0.6±1.1	18.5±3.6
12–13	12	160.0±5.7	0.4±0.6	46.2±7.0	0.8±0.7	20.0±2.8
13–14	16	157.0±7.0	0.1±0.9	44.7±6.4	0.1±0.8	18.1±1.8
14–15	17	165.0±4.6	0.4±0.7	49.2±3.9	0.1±0.5	18.1±1.8
15–16	15	166.5±7.6	0.1±1.3	52.7±5.7	0.1±0.8	19.0+1.8
16–17	12	169.1±4.4	0.1±0.8	55.1±4.4	0.0±0.6	19.3±1.3
17–18	16	172.0±4.5	0.5±0.9	55.8±6.1	−0.2±1.0	18.9±2.1
18–19	10	170.6±4.8	0.2±0.9	60.0±6.6	0.4±1.1	20.5±1.5
**Girls**						
5–6	11	109.1±3.9	−0.4±1.0	17.0±1.0	−0.4±0.4	14.3±0.8
6–7	12	115.7±6.4	−0.4±1.3	19.5±2.9	−0.2±0.8	14.5±1.6
7–8	12	123.6±4.4	0.3±0.8	23.7±3.7	0.3±0.7	15.5±2.0
8–9	18	124.9±6.7	−0.4±1.1	24.5±4.3	0.0±0.7	15.6±1.5
9–10	13	132.9±5.8	0.1±0.9	30.9±6.7	0.4±0.8	17.3±2.6
10–11	15	144.4±5.4	0.6±0.7	36.8±5.1	0.5±0.6	17.6±1.7
11–12	11	150.5±4.2	1.0±0.6	39.5±5.8	0.5±0.8	17.4±2.4
12–13	18	151.3±6.5	0.1±1.2	41.1±6.1	0.1±0.7	17.9±2.0
13–14	16	155.5±5.6	0.4±1.1	44.5±5.5	0.2±0.8	18.4±2.4
14–15	13	157.7±3.8	0.5±0.8	49.2±5.9	0.5±0.9	19.8±1.9
15–16	12	156.9±4.7	0.1±0.9	49.7±6.1	0.4±1.0	20.1±2.1
16–17	17	157.1±4.1	0.1±0.8	48.7±5.5	0.1±0.9	19.7±1.9
17–18	12	157.5±4.3	0.1±0.9	49.9±5.0	0.2±0.8	20.2±2.1
18–19	13	157.7±6.7	0.2±1.4	50.4±4.3	0.3±0.7	20.4±2.6

### BMDLS, BMDTB and BMADLS for different age groups

**Figure 1:**
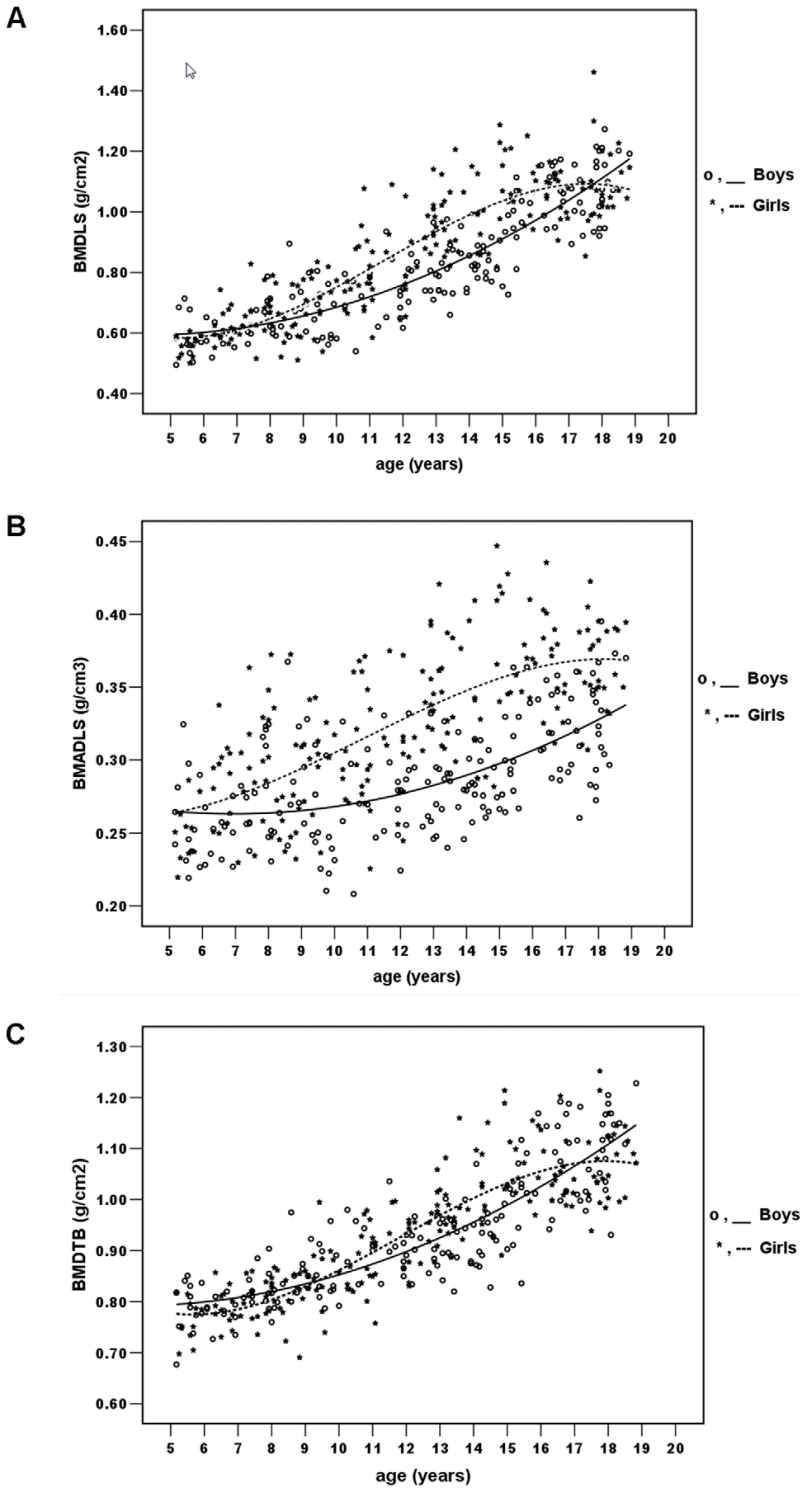
Relationships between age vs. BMD of the lumbar spine (BMDLS; grams per cm^2^), BMAD of the lumbar spine (BMADLS; grams per cm^3^) and BMD of the total body (BMDTB; gram per cm^2^) among boys and girls. The lines show the best- fitted function with the factors age, age^2^ and age^3^ (cubic function) for girls and age and age^2^ (quadratic function) for boys.

The reference data of BMDLS, BMADLS and BMDTB for boys and girls are shown in Table 2. The BMDLS, BMADLS and BMDTB values increased with age in both genders (Fig. 1). The maximal increase in the BMDLS, BMDTB and BMADLS occurred at the age of 11–12 years in girls and 18–19 years in boys. In girls, the age-dependent increase in BMDLS, BMADLS and BMDTB values leveled off after the age of 15–16 years, whereas in boys, BMDLS, BMADLS and BMDTB values continued to increase until the age of 18–19 years.

**Table 2:** Mean bone mineral density of the lumbar spine (BMDLS), bone mineral apparent density of the lumbar spine (BMADLS) and bone mineral density of total body (BMDTB) with standard deviation (SD) in Thai children and adolescents (174 boys and 193 girls) for each age group.

Age (yrs)	n	BMDLS (g/cm^2^)		BMADLS (g/cm^3^)		BMDTB g/cm^2^)	
		Mean	SD	Mean	SD	Mean	SD
**Boys**							
5–6	12	0.588	0.070	0.259	0.032	0.791	0.051
6–7	10	0.592	0.040	0.248	0.017	0.779	0.029
7–8	12	0.648	0.058	0.283	0.029	0.829	0.036
8–9	12	0.671	0.088	0.277	0.041	0.834	0.055
9–10	14	0.666	0.085	0.261	0.036	0.858	0.050
10–11	6	0.683	0.102	0.265	0.043	0.898	0.075
11–12	10	0.743	0.099	0.278	0.028	0.900	0.055
12–13	12	0.778	0.089	0.280	0.032	0.905	0.041
13–14	16	0.804	0.088	0.278	0.026	0.918	0.053
14–15	17	0.846	0.058	0.286	0.018	0.936	0.055
15–16	15	0.960	0.116	0.305	0.034	1.013	0.086
16–17	12	1.077	0.084	0.322	0.026	1.086	0.088
17–18	16	1.046	0.108	0.314	0.032	1.067	0.064
18–19	10	1.127	0.098	0.340	0.035	1.137	0.082
**Girls**							
5–6	11	0.558	0.035	0.250	0.020	0.762	0.041
6–7	12	0.624	0.059	0.286	0.027	0.790	0.036
7–8	12	0.663	0.082	0.290	0.038	0.799	0.039
8–9	18	0.650	0.083	0.288	0.046	0.814	0.047
9–10	13	0.715	0.091	0.301	0.030	0.854	0.062
10–11	15	0.797	0.119	0.310	0.038	0.875	0.059
11–12	11	0.800	0.139	0.300	0.043	0.901	0.068
12–13	18	0.898	0.124	0.329	0.038	0.938	0.060
13–14	16	0.968	0.108	0.348	0.033	0.981	0.065
14–15	13	1.007	0.150	0.346	0.055	1.043	0.100
15–16	12	1.089	0.102	0.375	0.034	1.059	0.058
16–17	17	1.067	0.071	0.366	0.032	1.049	0.059
17–18	12	1.082	0.160	0.366	0.035	1.061	0.092
18–19	13	1.092	0.077	0.367	0.024	1.087	0.059

The BMDLS values of girls aged 12 to 16 years and the BMDTB of girls aged 13 to 15 years were higher than in boys at the same age groups (*P*<0.01). Girls had higher BMADLS than boys at all age groups (*P*<0.05) except age groups of 5–6 years, 7–8 years and 8–9 years where no statistical difference in BMADLS was seen.

### BMCLS, BMCTB, BALS, BATB and LBM for different age groups

**Figure 2:**
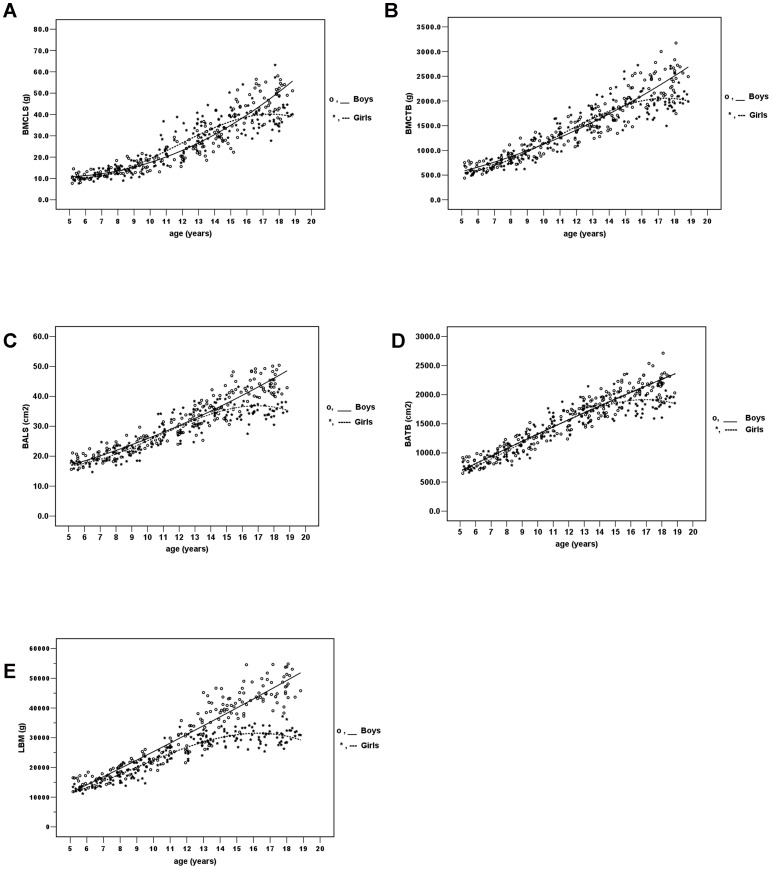
Relationships between age vs. BMC of the lumbar spine (BMCLS; grams), BMC of the total body (BMCTB; grams), BA of the lumbar spine (BALS; cm^2^), BA of the total body (BATB; cm^2^) and lean body mass (LBM; grams) among boys and girls. The lines show the best- fitted function with the factors age, age^2^ and age^3^ (cubic function) for girls and age and age^2^ (quadratic function) for boys.

Table 3 shows BMCLS, BALS, BMCTB, BATB and LBM values of boys and girls for each age group. BMCLS, BMCTB, BALS, BATB and LBM values progressively increased with age (Fig. 2).

**Table 3:** Mean and standard deviation (SD) of bone mineral content of lumbar spine (BMCLS), bone area of lumbar spine (BALS), bone mineral content of total body (BMCTB), bone area of total body (BATB) and lean body mass values (LBM) in Thai children and adolescents (174 boys and 193 girls) for each age group.

Age (yrs)	n	BMCLS (g)		BALS (cm^2^)		BMCTB(g)		BATB (cm^2^)		LBM (g)	
		Mean	SD	Mean	SD	Mean	SD	Mean	SD	Mean	SD
**Boys**											
5–6	12	10.8	2.0	18.3	2.0	653.2	106.1	821.8	97.3	14927	1877
6–7	10	11.8	1.4	19.9	1.4	671.9	84.7	861.3	94.8	15861	1701
7–8	12	13.3	2.1	20.5	2.3	835.6	124.9	1006.0	121.1	17897	1969
8–9	12	15.2	2.5	22.7	2.0	926.4	152.3	1107.6	130.2	20089	2141
9–10	14	16.5	2.7	24.8	2.3	1052.9	157.9	1223.2	139.7	22183	2166
10–11	6	17.0	2.9	24.8	0.8	1167.5	173.4	1295.5	101.8	22904	2782
11–12	10	21.4	4.5	28.6	2.8	1359.5	214.6	1507.3	190.5	28424	4071
12–13	12	24.1	3.8	30.9	2.2	1518.9	176.8	1674.9	147.7	32456	4201
13–14	16	27.2	5.7	33.6	4.2	1580.4	223.5	1716.4	182.6	36489	6697
14–15	17	31.0	4.4	36.5	3.3	1766.9	219.8	1882.9	147.4	40452	2704
15–16	15	38.4	8.5	39.6	5.2	2060.7	361.4	2019.5	215.5	42636	5823
16–17	12	47.9	6.4	44.3	3.7	2333.2	286.5	2143.3	123.7	45764	3134
17–18	16	46.4	6.0	44.3	3.2	2315.1	272.0	2166.5	174.5	45624	4761
18–19	10	50.1	5.8	44.4	3.5	2586.1	361.0	2267.8	222.9	49243	4151
**Girls**											
5–6	11	9.7	0.9	17.4	1.0	560.7	57.2	734.5	48.7	12873	907
6–7	12	11.4	1.9	18.2	1.7	670.7	102.1	847.1	109.7	14381	1246
7–8	12	13.8	2.0	20.8	2.0	781.0	109.3	974.7	103.4	16697	1546
8–9	18	13.5	2.2	20.8	2.2	834.7	124.8	1023.9	128.0	17780	2348
9–10	13	16.2	3.3	22.5	2.4	1049.5	207.5	1222.4	185.0	19985	2930
10–11	15	21.9	6.0	27.1	3.6	1250.3	222.1	1421.6	167.9	24198	2490
11–12	11	24.1	6.7	29.7	3.2	1406.5	240.6	1551.6	169.1	27374	3483
12–13	18	28.3	7.2	31.1	4.3	1532.2	253.1	1626.7	192.3	27641	2928
13–14	16	31.8	5.4	32.7	2.9	1710.5	217.4	1740.6	152.5	29763	1837
14–15	13	36.2	6.0	35.9	2.5	1899.6	371.6	1808.6	196.4	30715	2329
15–16	12	38.7	6.4	35.4	3.6	2018.0	270.9	1901.3	179.1	30624	2632
16–17	17	38.2	4.8	35.8	3.5	1941.4	239.5	1846.2	151.0	30509	2674
17–18	12	39.9	10.2	36.5	4.2	2007.1	343.3	1880.0	169.0	29860	2864
18–19	13	40.2	4.6	36.8	3.3	2102.9	196.2	1936.3	153.9	31504	2091

Mean BMCLS values were similar between both genders until the age of 12 years where BMCLS values for girls were higher than boys till the age of 16 years (*P*<0.05). Thereafter, BMCLS values of boys were higher than those of girls. Mean BALS were similar between both genders until the age of 15 years where BALS values for boys were higher than girls. (*P*<0.05).

For BMCTB and BATB values, boys had higher BMCTB and BATB values than girls starting at the age of 16 years (*P*<0.01).

The most apparent gain in BMC occurred during ages 10 to 14 years in girls and ages 15 to 19 years in boys, which was the time of their pubertal growth spurt. While BA increased steadily according to age. The magnitude of the increase of BMC was more pronounced among boys. Girls reached their peak BMC and BA at 16–17 years of age, while among boys, BMC and BA values continued to increase until the age of 18–19 years.

LBM values for boys were significantly higher than girls in almost all age groups (*P*<0.05). LBM increased rapidly during the pubertal growth spurt in both genders.

### BMDLS and BMDTB for each Tanner stage

**Figure 3:**
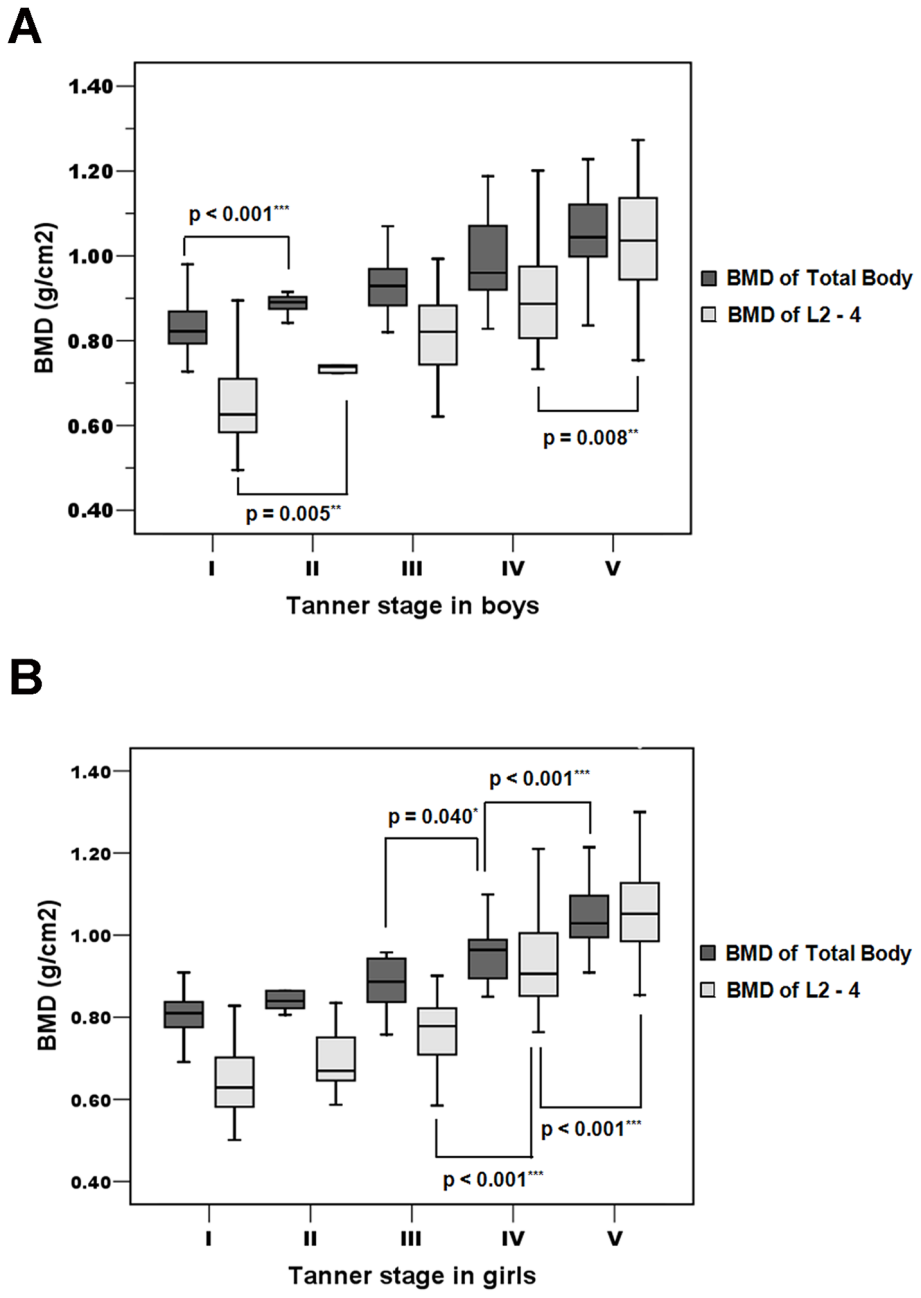
BMD of the lumbar spine (BMDLS; grams per cm^2^) and total body (BMDTB; gram per cm^2^) at different Tanner stages among boys and girls. ^*^P<0.05, **P<0.01, ***P<0.001 compared with previous Tanner stage.

Table 4 demonstrates BMDLS and BMDTB values for each Tanner stage in both boys and girls. Both BMDLS and BMDTB values increased with pubertal progression in both genders. A significant increase in BMDLS and BMDTB values compared to previous Tanner stage were observed in girls during late puberty (Tanner V and Tanner IV; *P*<0.001 both for BMDLS and *P*<0.001 and *P*<0.05 for BMDTB, respectively, Fig. 3B). In contrast, for boys, a significant increase was seen at Tanner II and V for BMDLS (*P*<0.01) and at Tanner II for BMDTB values (*P*<0.001) as shown in Fig. 3A. When comparing mean BMDLS and BMDTB values between boys and girls for each Tanner staging, no significant differences were observed.

**Table 4:** Mean and standard deviation (SD) of bone mineral density of the lumbar spine (BMDLS) and total body (BMDTB) in Thai children and adolescents (174 boys and 193 girls) for each Tanner stage.

Tanner stage	n	Boys		n	Girls	
		BMDLS (g/cm^2^)	BMDTB (g/cm^2^)		BMDLS (g/cm^2^)	BMDTB (g/cm^2^)
**I**	**72**	0.652	0.837	**67**	0.646	0.805
		(0.090)	(0.066)		(0.084)	(0.049)
**II**	**13**	0.736**	0.891***	**8**	0.694	0.857
		(0.065)	(0.032)		(0.079)	(0.059)
**III**	**20**	0.814	0.931	**10**	0.764	0.883
		(0.104)	(0.065)		(0.095)	(0.065)
**IV**	**21**	0.913	0.992	**33**	0.927***	0.961*
		(0.130)	(0.106)		(0.114)	(0.074)
**V**	**48**	1.033**	1.052	**75**	1.057***	1.046***
		(0.123)	(0.097)		(0.115)	(0.077)

^*^P<0.05, **P<0.01, ***P<0.001 compared with previous Tanner stage.

### The relationship between weight, height and pubertal status and BMDLS, BMDTB, BMADLS

The relationships between weight, height and Tanner stage vs. BMDLS, BMDTB and BMADLS values adjusted for age were assessed by multiple regression analysis. For boys, weight (*P*<0.05) and Tanner stages (*P*<0.05) were significantly related to BMDLS, BMDTB or BMADLS (R^2^ = 84%, 82% and 40%, respectively). For girls, weight (*P*<0.001) and Tanner stages (*P* = 0.026) were also significantly related to BMDLS (R^2^ = 84%). For BMDTB and BMADLS values (R^2^ = 82% and 57%, respectively), weight (*P*<0.01), Tanner stages (*P*<0.05) and height (*P*<0.05) were the significant determinants.

### Age of menarche and BMD

Fifty percent of girls in the study had already experienced menarche (the mean age of menarche was 12.2±1.3 years). For girls aged 12–14 years, BMDLS, BMDTB and BMADLS values were significantly higher in those who had experienced menarche than girls who had not (*P*<0.001, 0.011 and <0.001, respectively).

### The amount of daily calcium intake and BMD

The average calcium intake was 918±497 mg/day. The amount of daily calcium intake among boys and girls was not significantly different (977±557 vs. 866±433 mg/day, *P* = 0.065). There were no relationships between daily calcium intake and age, BMDLS, BMDTB or BMADLS values in boys or girls (data not shown).

## Discussion

A normative reference of BMD for specific ethnicity is crucial for interpretation of bone health status. Differences in BMD among ethnicities as measured by DXA have been reported. BMD values of Chinese and Japanese individuals are lower than that of Caucasians [21]. Also, African American men have higher BMD than Caucasian men [22]. Therefore, to accurately assess BMD, ethnic-specific normative BMD values are necessary. Normative BMD data for children are available for Caucasians from different parts of the world including US, Spanish, Dutch and Swedish children [10], [11], [12], [13].

However, BMD references of Asian children are limited; only Japanese, Chinese, Korean and Indian data are available and none from Southeast Asian population [14], [15], [16], [17]. Our study is the first study to provide the normative reference data for BMDLS, BMDTB, BMADLS, BMCLS, BMCTB, BALS, BATB and LBM values measured by DXA (Lunar Prodigy) in healthy Thai children and adolescents aged 5–18 years.

**Figure 4:**
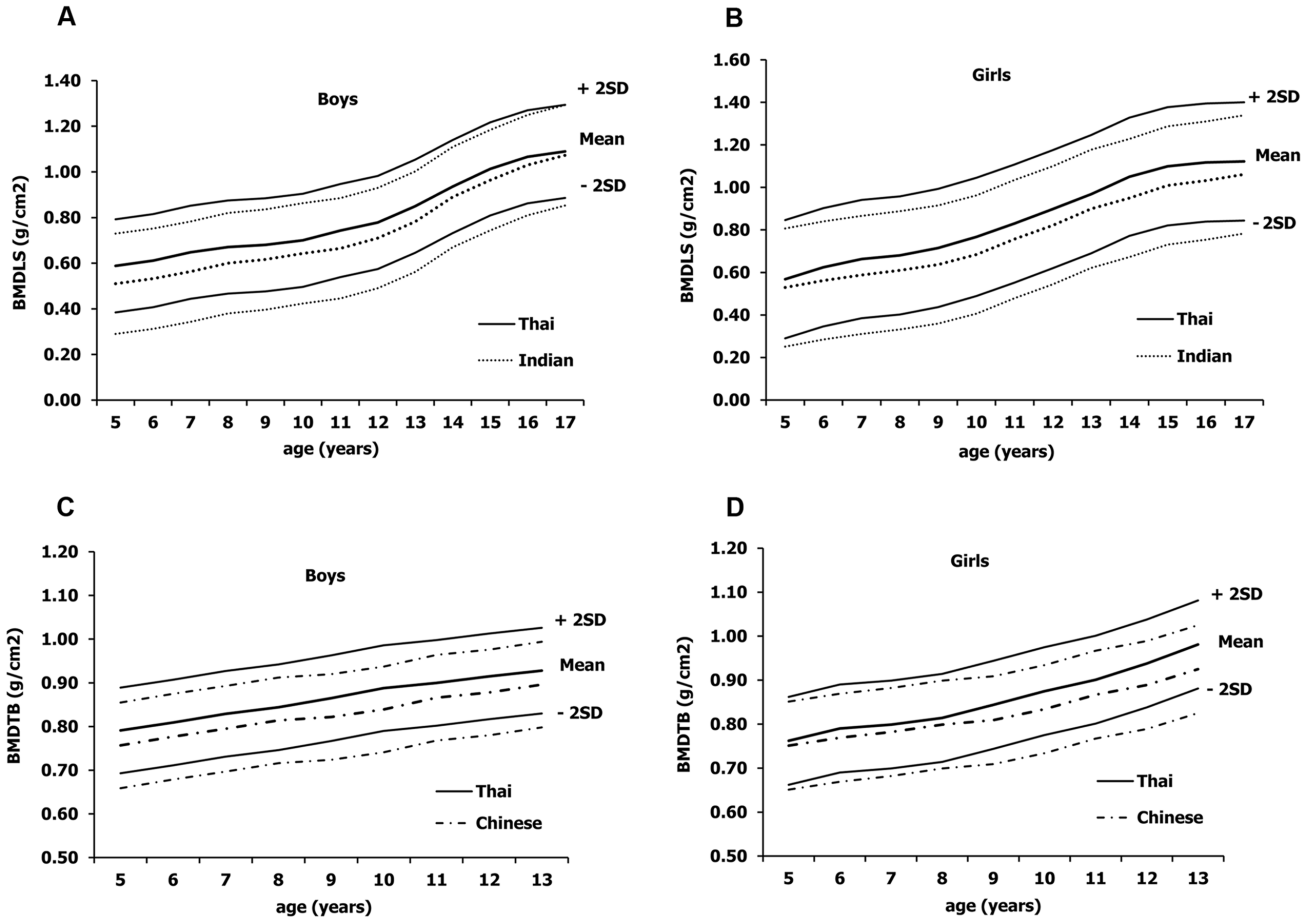
Comparison between our current Thai vs. Indian and Chinese BMD normative data in children and adolescents among boys and girls. These figures were drawn based on data of the mean ± 2SD available from references [15], [17]. The lines show mean ± 2SD. Thai BMDLS values are higher than those of Indian children and adolescent for both genders. Comparing to Chinese children, Thai children have higher BMDTB values for both genders.

Oldroyd et al previously reported that reference data obtained from pencil-beam densitometers (Lunar DPXL) can be used as reference data for fan-beam densitometers (Lunar Prodigy) since there were no significant differences in the lumbar spine, femoral neck and total body BMD measured by these two different DXA platforms [23]. We therefore compared our BMD data with those of published data using DXA from the same vendor (Prodigy or DPX-L, Lunar Corp., Madison, WI, USA). In general, Thai children have lower mean BMDLS (6%) than Caucasian [24]. On the contrary, our mean BMD reference values are 8% and 4% higher than those of Indian (N = 920; BMDLS) and Chinese children (N = 877, BMDTB), respectively [15], [17]. These differences among Asian ethnicity are demonstrated in Figure 4 comparing data between Thai vs. Indian and Chinese children. The differences of our BMD normative data from the Indian and Chinese populations might reflect on variation of daily calcium intake, nutritional status, physical exercise, daily activities, vitamin D levels and the amount of sun-exposure per year. For example, the prevalence of vitamin D deficiency was 5.7% in Thailand [25] while it was approximately 90% and 40% in India and China, respectively [26], [27]. Differences of these contributing factors on bone health and BMD parameters further highlight the necessity of using a population-specific normative data even among Asian children living in various subcontinents.

Similar to previous reports, the dynamic changes of BMD values from childhood to early and late puberty of Thai children appeared to be consistent with those of Caucasian and Asian populations; BMDLS, BMDTB and BMADLS increase with age [10], [11], [12], [13], [15], [16], [17]. A progressive increase of BMADLS also indicates an actual raise in mineralization, rather than an enlargement in the bone size during growth [28]. In addition, the BMDLS and BMDTB values also increase according to the pubertal progression and there was no difference between boys and girls at the same pubertal stage [10], [11], [12], [16], [17]. This finding indicates that the observed higher BMDLS and BMDTB values in girls; aged 12–16 years was mainly due to earlier puberty in girls. Furthermore, higher Tanner stage including menarche in girls were found to be directly associated with increase on BMDLS, BMDTB and BMADLS. Taken together, these findings emphasize the importance of sex steroids as it acts in concert with growth hormone to increase BMD during puberty [29].

The pattern of increase in BMC and BA with age is similar to those of BMD. The rapid increase in BMC was paralleled with BMD during the pubertal growth spurt similar to previous studies [12], [13], [16], [17]. The BMC depends on the amount of mineral deposit in bone matrix, bone size and the height of subjects. Therefore, BMC should be adjusted by the surface area scanned and expressed as BMD values in growing children [12].

In addition, the positive correlation between BMD and several demographic parameters, except the calcium intake, was also observed in our study, similar to what has been reported [10], [16]. For example, weight and Tanner stages in both boys and girls have significant influence on BMDLS, BMDTB and BMADLS values independent of age. In addition, height also has a significant influence on BMDTB and BMADLS in girls. For calcium intake, adequate consumption is essential for bone mass acquisition and for reaching peak bone mass [30]. Boot et al previously reported a positive correlation between the calcium intake and BMDTB in boys [10]. We did not find such an association in the present study in both genders. Given the complexity of the average Thai meal, subjects may have difficulty recalling their consumption accurately therefore the calculated daily calcium consumption might not be correct. Future studies should utilize a daily food record rather than a one-time questionnaire dependent on patients' recalls.

Besides BMD, BMC and BA measurement, DXA can provide additional data on the lean body mass (LBM). Our study described normative LBM values representing mainly muscle mass for both boys and girls and showed that this parameter increased with age and was mainly higher in boys than girls at each age group similar to those of Caucasian and Asian populations [13], [17].

The correct BMD interpretation is critical for the diagnosis and management of children with suspected low bone mass. Since BMD measured by DXA is calculated as BMC per a two-dimensional projected bone area, not a true bone volume, therefore in clinical practice BMD values of children should be adjusted for their sizes. Questions of the validity of the use of areal BMD as a substitution for volumetric BMD have been raised. Various mathematical methods have been created to correct for bone size. At the present, there is no consensus on which method is the most appropriate for correction of bone size [31].

For example, Mølgaard et al have constructed the centile curves for bone area for age, BMC for age, bone area for height and BMC for bone area by using the LMS method and proposed that bone mineralization should be assessed based on height for age, bone area for height and BMC for bone area [32]. Crabtree et al created equations which account LBM as a predictor of BMC [33]. However, these two methods are technically challenging for daily clinical practice.

In our study, we have taken the differences in bone sizes into the account by calculating the apparent BMAD of the lumbar spine (BMADLS) values by using the method derives bone volume based on bone area data obtained from DXA [20].

There are other practical and simple methods for size adjustment for pediatrician to evaluate pediatric bone health which are BMD adjusted for height age (HA) and BMD adjused for bone age (BA) [34], [35]. Both methods have been used for the BMD evaluation in short children with various diseases such as chronic kidney disease, thalassemia and Fanconi anemia [36], [37], [38].

One of the limitations of our study was that only six boys were recruited at the age group of 10–11 years. However, the standard deviations of BMD, BMADLS, BMC, BA, and LBM were modest and comparable with other age groups. Moreover, the data were fairly distributed. Therefore the reference data for boys at this particular age group should be acceptable. Another limitation of the present study was that the subjects were recruited only from Bangkok representing mainly urban population. However, recent study suggests that due to the current improvement in standard of living and health care in Thailand in the past 20 years, baseline nutritional status and physical growth do not differ significantly between urban and rural Thai children [39]. Therefore, it is quite likely that our normative reference could be of useful for clinical application nationwide.

In summary, we report the normative data of BMD, BMADLS, BMC, BA and LBM measured by DXA (Lunar, Prodigy) in Thai children and adolescents aged 5 to 18 years. Considering the similarity of geographical location, genetic background, the level of health development including nutritional status and daily lifestyle among Thai and other Southeast Asian children, our reference data will be of useful for clinicians and researchers to assess BMD status in Southeast Asian children. Size-adjusted method should be used for the interpretation of BMD measured by DXA in children with growth disorders.
